# Community-based surveys for *Plasmodium falciparum pfhrp2* and *pfhrp3* gene deletions in selected regions of mainland Tanzania

**DOI:** 10.1186/s12936-020-03459-3

**Published:** 2020-11-04

**Authors:** Catherine Bakari, Sophie Jones, Gireesh Subramaniam, Celine I. Mandara, Mercy G. Chiduo, Susan Rumisha, Frank Chacky, Fabrizio Molteni, Renata Mandike, Sigsbert Mkude, Ritha Njau, Camelia Herman, Douglas P. Nace, Ally Mohamed, Venkatachalam Udhayakumar, Caleb K. Kibet, Steven G. Nyanjom, Eric Rogier, Deus S. Ishengoma

**Affiliations:** 1 Jomo Kenyatta University of Agriculture and Technology, Nairobi, Kenya; 2 National Institute for Medical Research, Tanga Research Centre, Tanga, Tanzania; 3 Malaria Branch, Division of Parasitic Diseases and Malaria, Centers for Disease Control and Prevention, Atlanta, GA USA; 4 Williams Consulting, Baltimore, MD USA; 5 Oak Ridge Institute for Science and Education, Atlanta, GA USA; 6 Kilimanjaro Christian Medical University College, Moshi, Tanzania; 7 National Institute for Medical Research, Dar es Salaam, Tanzania; 8 National Malaria Control Programme (NMCP), Dodoma, Tanzania; 9 World Health Organization (WHO) Country Office, Dar es Salaam, Tanzania; 10 CDC Foundation (CDCF), Atlanta, GA USA; 11 Faculty of Pharmaceutical Sciences, Monash University, Melbourne, Australia; 12 Harvard T.H Chan School of Public Health, Boston, MA USA

**Keywords:** Tanzania, Malaria, Rapid diagnostic tests, Histidine-rich protein 2/3, Lactate dehydrogenase, Aldolase, *Plasmodium falciparum*

## Abstract

**Background:**

Histidine-rich protein 2 (HRP2)-based malaria rapid diagnostic tests (RDTs) are effective and widely used for the detection of wild-type *Plasmodium falciparum* infections. Although recent studies have reported false negative HRP2 RDT results due to *pfhrp2* and *pfhrp3* gene deletions in different countries, there is a paucity of data on the deletions of these genes in Tanzania.

**Methods:**

A community-based cross-sectional survey was conducted between July and November 2017 in four regions: Geita, Kigoma, Mtwara and Ruvuma. All participants had microscopy and RDT performed in the field and provided a blood sample for laboratory multiplex antigen detection (for *Plasmodium* lactate dehydrogenase, aldolase, and *P. falciparum* HRP2). Samples showing RDT false negativity or aberrant relationship of HRP2 to pan-*Plasmodium* antigens were genotyped to detect the presence/absence of *pfhrp2/3* genes.

**Results:**

Of all samples screened by the multiplex antigen assay (n = 7543), 2417 (32.0%) were positive for any *Plasmodium* antigens while 5126 (68.0%) were negative for all antigens. The vast majority of the antigen positive samples contained HRP2 (2411, 99.8%), but 6 (0.2%) had only pLDH and/or aldolase without HRP2. Overall, 13 samples had an atypical relationship between a pan-*Plasmodium* antigen and HRP2, but were positive by PCR. An additional 16 samples with negative HRP2 RDT results but *P. falciparum* positive by microscopy were also chosen for *pfhrp2/3* genotyping. The summation of false negative RDT results and laboratory antigen results provided 35 total samples with confirmed *P. falciparum* DNA for *pfhrp2/3* genotyping. Of the 35 samples, 4 (11.4%) failed to consistently amplify positive control genes; *pfmsp1* and *pfmsp2* and were excluded from the analysis. The *pfhrp2* and *pfhrp3* genes were successfully amplified in the remaining 31 (88.6%) samples, confirming an absence of deletions in these genes.

**Conclusions:**

This study provides evidence that *P. falciparum* parasites in the study area have no deletions of both *pfhrp2* and *pfhrp3* genes. Although single gene deletions could have been missed by the multiplex antigen assay, the findings support the continued use of HRP2-based RDTs in Tanzania for routine malaria diagnosis. There is a need for the surveillance to monitor the status of *pfhrp*2 and/or *pfhrp3* deletions in the future.

## Background

Upon successful establishment of blood-stage infection by *Plasmodium* parasites*,* various parasite proteins are produced and released into the host blood*.* Some of these proteins (also referred to as antigens) are targets for malaria rapid diagnostic tests (RDTs). Three antigen targets currently in use include *Plasmodium* lactate dehydrogenase (pLDH), *Plasmodium* aldolase (aldolase), and the *Plasmodium falciparum*-specific histidine rich protein 2 (HRP2) [1–3]. The use of antigen-based RDTs in many malaria-endemic countries worldwide have profoundly improved malaria case management and surveillance efforts and remains an essential diagnostic tool, especially in Africa [4–7].

HRP2-based tests are species-specific since the antigen is only produced by *P. falciparum*, though tests detecting pLDH and aldolase have the potential to detect all human malarias [2, 4, 8]. HRP2 is the most widely used antigen in RDTs either alone or in combination with other antigens, due to its abundance, specificity for *P. falciparum* infection, and high sensitivity and thermal stability [9]. However, antibodies raised against HRP2 can cross-react with *P. falciparum* HRP3 antigen due to similarities in amino acid sequences and repeating epitopes [10–12]. The genes encoding for these two antigens are located on different chromosomes of the *P. falciparum* genome, with *pfhrp2* on chromosome 8 while *pfhrp3* gene is on chromosome 13 [13, 14]. A large number of parasites with genetic deletions of *pfhrp2* and/or *pfhrp3* genes in natural populations of *P. falciparum* were first reported in Peru and subsequently in other countries including in Africa with potential negative impacts on the performance of currently used RDTs [15–22].

Sensitivity of HRP2-based RDTs can be affected by transportation and storage conditions outside of manufacturer specifications, operator errors, low density infections, or a mutation or deletion of the *pfhrp2* and/or *pfhrp3* genes in the infecting parasite strain [15–23]. In addition, the diversity in parasite population and number of epitopes on HRP2 recognized by the diagnostic test antibodies may modify the sensitivity of the test when dealing with different *P. falciparum* populations [24–28].

In Tanzania, RDTs were introduced between 2009 and 2012 and are now widely used in both private and public health facilities throughout the country. Before the introduction of RDTs in Tanzania, a study conducted between 2005 and 2010 (the African Quinine Artesunate Malaria Treatment Trial) found no evidence of *pfhrp*2*/*3 gene deletions [29]. However, *pfhrp*2 gene deletions have been reported in the neighbouring East African countries, including Kenya [21] and Rwanda [27]. In addition, two studies conducted in Tanzania which analysed samples collected in 2010, and between 2016 and 2018 showed evidence of sporadic occurrence of *pfhrp*2*/*3 gene deletions in some areas [30, 31]. Although both the initial evidence (sample confirmed as microscopy positive *for P. falciparum* but negative PfHRP2-detecting RDTs) and confirmatory evidence (molecular approaches) [32] were used to screen for *pfhrp*2/3 gene deletions, the sample size and the geographic regions covered were limited. In this study, field diagnostic results and a multiplex antigen detection assay were used to investigate potential *pfhrp*2/3 gene deletions in samples collected in 2017 from four regions of Tanzania with persistently high malaria transmission after five years of introduction of RDTs.

## Methods

### Study sites

**Fig. 1:**
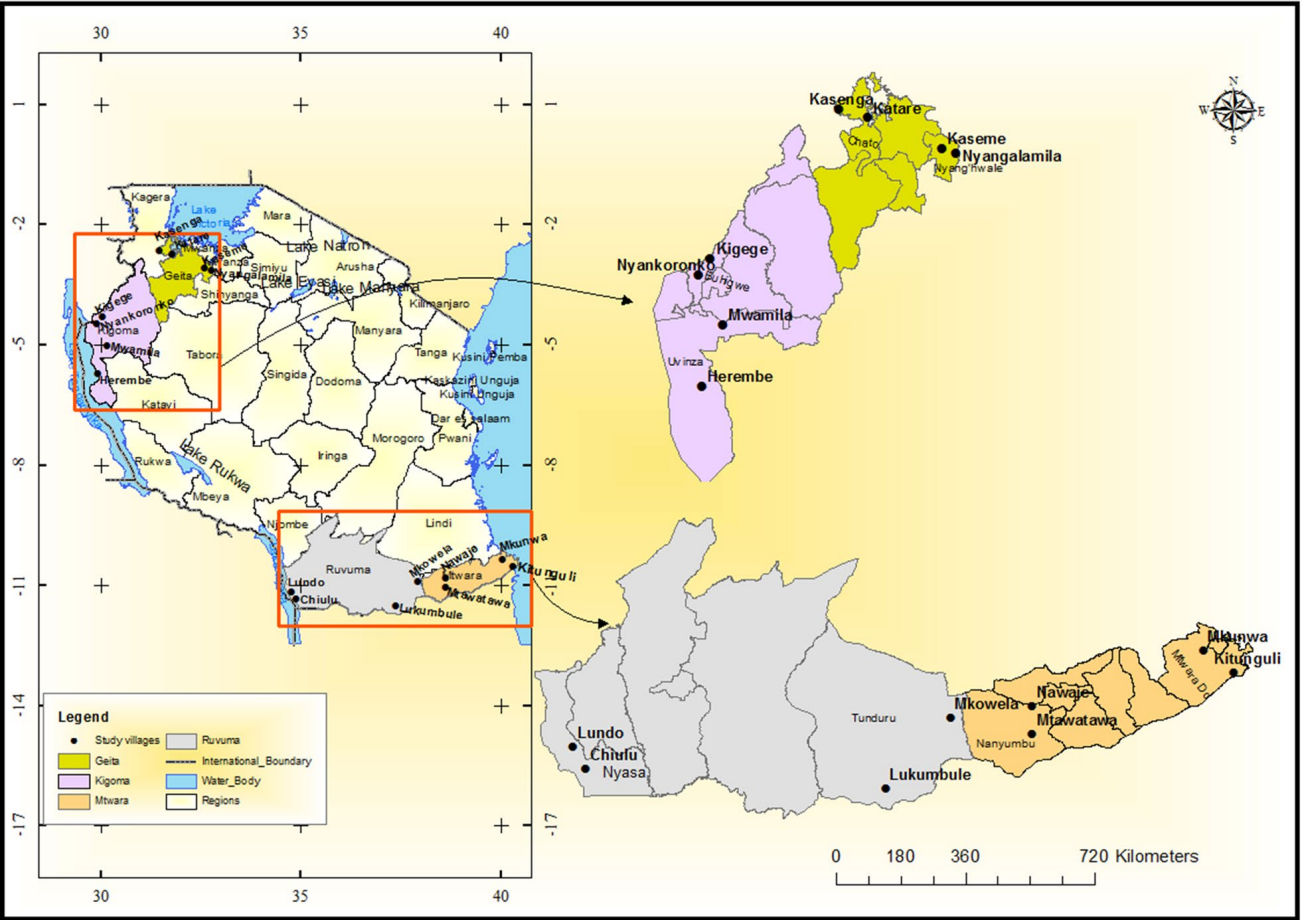
Map showing the study sites in the four regions of Tanzania

Samples and data were obtained from a cross-sectional community survey (Hotspots study) involving mainly asymptomatic individuals which was conducted between July and November 2017 in four regions of Tanzania (Geita, Kigoma, Mtwara and Ruvuma) [33]. These regions were among those with persistently high malaria transmission as shown by the surveys conducted from 2007 to 2017 [34–37]. The four Regions also had higher prevalence in the School Malaria Parasitological Survey (SMPS) of 2014/2015 [38], and are among the 10 regions targeted by the National Malaria Control Programme (NMCP) for reduction of malaria burden through the high burden to high impact initiative (based on WHO and NMCP revised strategic plan). Two districts with high prevalence in the SMPS of 2014/2015 were purposively selected from each region; Nyang’hwale and Chato (Geita), Buhigwe and Uvinza (Kigoma), Mtwara DC and Nanyumbu (Mtwara) and Nyasa and Tunduru (Ruvuma). Within each district, two villages were purposively selected for sampling based on the malaria parasite positivity rates as reported from health facility reports, making a total of 16 villages sampled (Fig. 1).

In each of the sampled villages, a random sample of at least 120 households (HHs) were selected and all members of these HHs were asked to participate in the survey [33]. Blood samples were collected by finger prick, thin and thick films were prepared, and all study participants were screened with malaria RDTs as per the manufacturer’s instructions. Care Start Malaria HRP2/pLDH (Pf/PAN) COMBO (AccessBio, NJ, USA) RDTs were used in Geita and Kigoma regions, and Lundo village of Nyasa District (Ruvuma region). The RDTs were depleted due to testing a large number of community members who sought clinical care and were replaced with Care Start Malaria HRP2 (Pf) (AccessBio, NJ, USA), which were readily available from suppliers. These RDTs [Care Start Malaria HRP2 (Pf) (AccessBio, NJ, USA)] were used in the rest of the villages in Ruvuma (2 in Tunduru district) and Mtwara regions (4 villages, two in each of districts of Nanyumbu and Mtwara DC). The results were interpreted within the specified reading time of the manufacturer’s protocol. Dried blood spots (DBS) on filter papers were collected on Whatman 3MM paper (GE Healthcare, PA, USA), dried for 2–4 h, and individually packaged in sealable plastic bags with desiccant for further laboratory analysis. Participants with RDT positive results were treated according to the national guidelines [39].

The main study and the laboratory analyses reported in this manuscript obtained ethical approval from the Medical Research Coordinating Committee (MRCC) of the National Institute for Medical Research (NIMR), and permission to conduct the study in the selected regions was sought from the President’s Office, Regional Administration and Local Government Authority and Regional, District and village authorities. Informed consent/assent was sought before conducting the demographic survey or including participants into the parasitological part of the study. A written informed consent for laboratory analyses including detection of *pfhrp2/3* gene deletion which was performed in this study was obtained from the sample donors. The laboratory activities undertaken at CDC were considered non-research by the CDC Human Subjects office for the purpose of providing laboratory testing of these specimens and participation of CDC scientists for this collaboration.

### Microscopy

Thick and thin blood films for parasite counting and species identification were prepared from the finger prick blood and stained using 3% Giemsa for 45 min to detect parasite infection status and parasite density using thick films while parasite species were assessed on thin films. Parasites were counted as asexual parasites per 200 White Blood Cells (WBCs) for asexual parasites or 500 WBCs for sexual stages. A blood film was declared negative if no *Plasmodium* parasites were detected after examining 200 high power fields for the thick film. Parasite density (parasites per µL of blood) was calculated by multiplying the number of asexual parasites by 40 or sexual stages by 16 assuming one microlitre of blood contained 8000 WBC. For the purposes of quality control, each blood smear was examined by two trained microscopists blinded of the RDT results. The final parasitaemia was taken as the average of the counts of the two microscopists if their results did not differ by > 50% for blood smears with ≥ 400 asexual parasites/μL of blood. In blood smears with < 400 asexual parasites/μL, any counts of each of the two microscopists was accepted and used to calculate the average parasitaemia. Blood smears with discordant results were re-examined by a third microscopists and the results of any two microscopists was accepted as explained above. Further discordant smears were resolved by a team of three microscopists who re-examined such smears at the same time based on previously-described protocol [40].

### Sample processing and laboratory multiplex assay

DBS were shipped to the Malaria Laboratory, at the Centers for Disease Control and Prevention, Atlanta, under ambient temperature. A 6 mm punch of each sample was taken and eluted in blocking buffer containing: PBS, 0.5% polyvinyl alcohol (Sigma, St. Louis, MO), 0.8% polyvinylpyrrolidine (Sigma), 0.1% casein (ThermoFisher Scientific, Waltham, MA), 0.5% BSA (Sigma), 0.3% Tween-20, 0.05% sodium azide, and 0.01% *E. coli* extract to prevent non-specific binding. The elution step diluted the samples to a 1:20 × whole blood dilution, which was the dilution used for the assay. DBS samples were screened by a bead-based multiplex antigen assay for the simultaneous detection of *P. falciparum* HRP2 (HRP2), pan-*Plasmodium* aldolase (aldolase), and pan-*Plasmodium* lactate dehydrogenase (pLDH) based on previously-described protocol [41]. Antibodies used to detect epitopes on HRP2 also targeted the same epitopes on the HRP3 antigen.

### DNA extraction, PET-PCR, *pfhrp2* and *pfhrp3* genotyping

A total of 94 samples with discordant results between microscopy and RDTs were selected for further molecular characterization. Genomic DNA was extracted from DBS of these 94 samples using QIAamp DNA Mini Kits (Hilden, Germany) using manufacturers’ protocol and screened for parasite DNA using the multiplex photo-induced electron transfer PCR (PET-PCR) assay as previously described [42–45]. PCR for *pfhrp2* and *pfhrp3* genotyping was performed as described previously [46, 47]. For genotyping of gene deletions, 3D7, Dd2 and HB3 DNA were used as controls for the assay; 3D7 as a positive control for both *pfhrp2* and *pfhrp3* while Dd2 was a negative control for *phrp2* but positive for *phrp3*, and HB3 was a negative control for *pfhrp3* but positive for *pfhrp2*. To confirm the absence of amplification events of the two genes, single copy *msp1* and *msp*2 genes were amplified. Complete details for the molecular assays are outlined in Additional file 1: Tables S1 and S2.

### Data analysis

The database and the different data collection applications were created using the Open Data Kit (ODK) software. Data cleaning, validation and quality control were undertaken as described by Chiduo et al. [33]. The data was later transferred to Microsoft Excel (Redmond, WA, USA) and STATA software (Texas, USA) for analysis which involved generating a summary of basic features of the study population.

To determine if a sample’s laboratory mean fluorescence intensity minus background signal (MFI-bg) was to be denoted as positive for a specific antigen, two methods were employed. First, a panel of 24 known negative blood samples which had been eluted from Whatman 903 filter paper were run by the multiplex antigen assay, the lognormal mean and standard deviation was derived from this sample set. The mean + 3sd was calculated to provide a MFI-bg threshold signal which was used as a cut-off to define antigen positive samples. Second, a two-component finite mixture model was used for the log-transformed antigen MFI-bg data from the study, and the mean + 3sd of the first component was used to define this cut-off. In order to reduce Type I errors, the more conservative of these two methods were used to determine the MFI-bg signal where any sample values above this would be considered a true positive for that particular antigen [48].

In comparing the HRP2 antigen signal to either of the pan-*Plasmodium* markers, the typical relationship between these two antigens was defined as the standard correlation observed for the vast majority of the observations. Visual outliers to this standard correlation were identified as outliers with suspicion of aberrant HRP2 production by the *P. falciparum* parasite requiring further molecular investigation (Fig. 3).

## Results

A total of 2520 out of 6207 registered HHs (40.6%) were sampled, with 7313 mainly asymptomatic individuals covered in the cross-sectional survey, which was conducted in 16 villages (in 8 districts) from four regions of Tanzania (Geita, Kigoma, Mtwara and Ruvuma) between July and November 2017 (Table 1). Apart from the 7313 individuals enrolled, an additional of 230 samples were taken from individuals who came to seek health services, but were not from the 120 sampled HHs. Therefore, 7543 blood samples were available for this study, and 3.0% (230/7543) of these were from individuals with incomplete data. The remainder (97.0%, 7313/7543) had complete data with parasitological, clinical and demographic information. The mean age of participants was 22.3 years (SD = 21.0) and 43.4% were male (Table 1).

**Table 1:** Baseline characteristics of individuals sampled in each of the four regions

	Mtwara	Geita	Kigoma	Ruvuma	Total
Number of HHs registered	1306	1209	1490	2202	6207
Number of HHs sampled n (%)	684 (52.4)	533 (44.1)	587 (39.4)	716 (32.5)	2520 (40.6)
Number of individuals in HHs	4723	6994	7945	8699	28,361
Number of individual sampled; n (%)	1548 (32.82)	2053 (29.4)	1979 (24.9)	1733 (19.9)	7313 (25.8)
Age in years; mean (SD)	25.3 (22.1)	18.2 (18.2)	22.4 (22.1)	24.3 (21.3)	22.3 (21.0)
Sex = Male; n (%)	702 (45.3)	878 (42.8)	850 (43.0)	742 (42.8)	3172 (43.4)
Microscopy positive; n (%)^a^	209 (13.5)	361 (17.6)	558 (28.2)	378 (21.8)	1506 (20.6)
RDTs positive n(%)^a^	553 (35.7)	556 (27.1)	737 (37.2)	591 (34.1)	2437 (33.3)
Fever^b^—Yes; n (%)^a^	245 (15.8)	261 (12.7)	736 (37.2)	275 (15.9)	1517 (20.7)
GMPD of positives; p/ul, (95% CI)	385 (299–495)	774 (643–931)	583 (482–706)	527 (435–638)	575 (516–637)

*HH* = Household, *SD* = standard deviation, *N* = number of individuals, *GMPD* = geometric mean parasite density, *CI* = Confidence interval

^a^*N* = 7313, ^b^ Fever within the past two weeks

For all enrolled participants, 38.4% (2897/7543) were positive for any one of the three parasite detection assays: microscopy, RDT, or bead-based multiplex assay. The results showed that 20.6% (1506/7313) of the participants were positive for *P. falciparum* infection by microscopy, 33.3% (2437/7313) were positive by HRP2 RDT, and 32.0% (2417/7543) were positive for anyone of the *Plasmodium* antigens tested by the bead-based multiplex assay. For those who were positive by microscopy, the geometric mean parasite density was 575 asexual parasites/µL of blood.

**Fig. 2:**
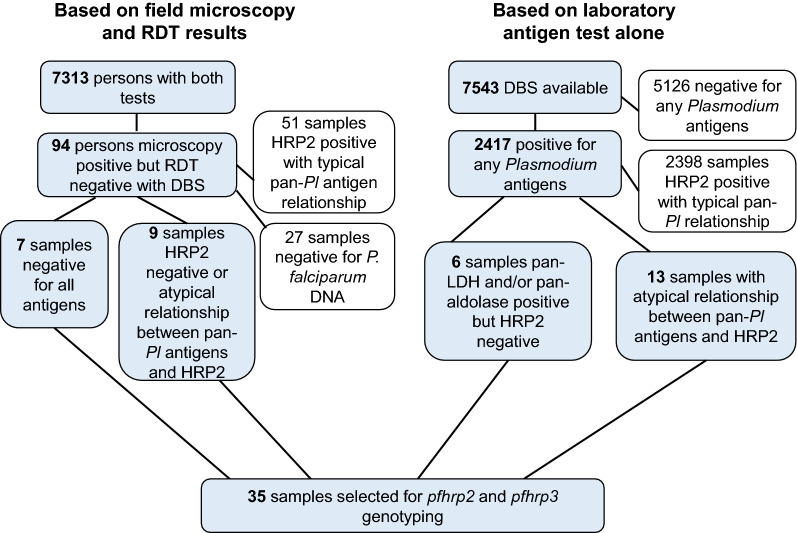
Flowchart for selection of samples requiring genotyping for *pfhrp2* and *pfhrp3*. Samples were selected based on initial field microscopy and RDT results (shown on left), or multiplex laboratory antigen detection (shown on right), for final determination of samples requiring genotyping to detect potential deletion of *pfhrp2/3* genes

**Fig. 3:**
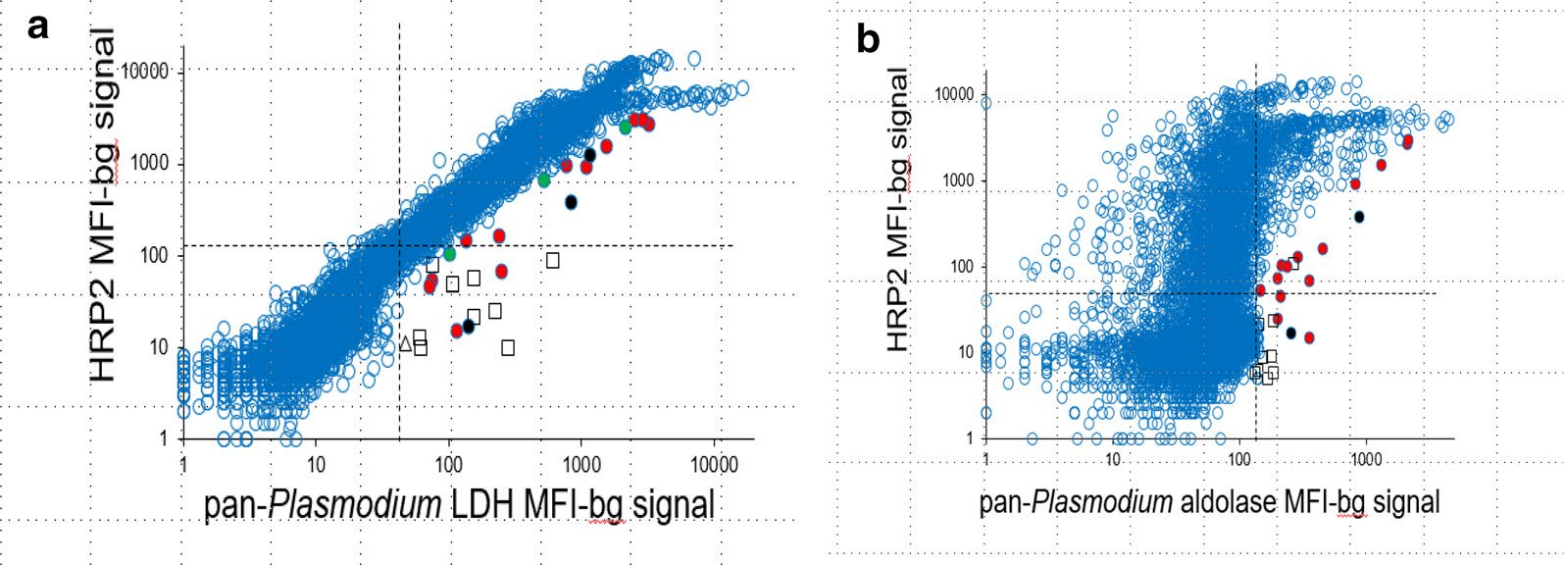
Scatterplots of pan-*Plasmodium* LDH or aldolase assay signal in comparison with HRP2 assay signal. Plots designate samples that were selected for further genotyping investigation as based on an atypical relationship to the pan-*Plasmodium* LDH (**a**) or aldolase (**b**) antigens. Hashed lines in each plot show MFI-bg assay signal threshold which would indicate a positive assay signal for each antigen. Black circles indicate samples selected based on field RDT results as well as laboratory antigen assay that were *P. falciparum* DNA positive. Red circles indicate samples selected solely based on laboratory antigen assay that were *P. falciparum* DNA positive. Squares indicate samples selected solely based on laboratory antigen assay that were *P. falciparum* DNA negative

A flow diagram for sample selection for further molecular testing to detect the presence (and potential prevalence) of *pfhrp2* and *pfhrp3* deletions in this study population is shown in Fig. 2. In selecting specimens warranting molecular assays for *pfhrp2* and *pfhrp3* genotyping, two types of information were considered: discordance between field microscopy and RDT results for an individual, and the relationship between the pan-*Plasmodium* antigens and the HRP2 antigen for an individual’s blood sample. From the field tests (microscopy and RDT), 95 persons were found to be microscopy positive but RDT negative (1.3% of the 7313 persons who had data for both tests). Of these 95 persons, 94 had a DBS available for multiplex antigen detection, and 54.3% (51/94) of these were found to be HRP2 antigen positive by bead-based multiplex assay with a typical relationship to the other pan-*Plasmodium* antigens. Due to the cross-reactivity nature of HRP2 and HRP3, the multiplex assay is non-discriminatory, therefore a positive signal would indicate presence of either HRP2 or HRP3 antigen or both antigens (as illustrated in Fig. 3). Additionally, 28.7% (27/94) of these samples were negative for *P. falciparum* DNA and could not undergo genotyping. The remaining 16 samples (17.0% of the 94 selected by microscopy/RDT discordance) were all *P. falciparum* DNA positive, but had no antigens detected (n = 7) or an atypical relationship between the pan*-Plasmodium* markers and HRP2 (n = 9). Based on these test results, these 16 were selected as warranting *pfhrp2* and *pfhrp3* genotyping.

Since all DBS were screened by the multiplex antigen assay, samples could be selected for *pfhrp2* and *pfhrp3* genotyping based solely on bead-based multiplex assay results. Of all 7543 DBS screened by the multiplex antigen assay, malaria antigen could not be detected in 5126 (68.0%) samples. Of the 2417 DBS positive for any antigens, 2398 (99.2%) of these were found to have a typical relationship of the pan-*Plasmodium* markers with HRP2 (Fig. 3). Of the remaining 19 DBS, which were all positive for the pan-*Plasmodium* antigens; 6 had a complete absence of HRP2 antigen, and 13 had an atypical relationship between the assay signal for the pan markers and HRP2. All of these 19 were positive for *P. falciparum* DNA and could thus be utilized for genotyping by PCRs. Together with the 16 samples chosen initially based on field test results, these 35 samples (16 + 19) were gathered as the final set with suspicion of aberrant HRP2 and/or HRP3 antigen production and warranted genotyping to confirm the absence/presence of *pfhrp2* and *pfhrp3* genes.

Table 2 outlines the *pfhrp2* and *pfhrp3* genotyping results for these 35 samples, as well as other information regarding the characteristics of the individuals, field test results, and other factors. Most of the samples (31/35, 88.6%) were found to successfully amplify *pfhrp2* and *pfhrp3* genes for the two exons of each gene. However, one or more of the *pfhrp2* and *pfhrp3* exon targets could not be amplified in 4 (11.4%) DNA samples. To correctly report the presence of a deletion (i.e. lack of PCR amplification), two other single-copy genes (*pfmsp1* and *pfmsp2*) were chosen and amplified to verify true non-amplification events [32]. For these 4 DNA samples, all were unable to consistently amplify both *pfmsp1* and *pfmsp2* single-copy genes and were excluded in the analysis. For this reason, non-amplification of *pfhrp2* and *pfhrp3* targets due to true deletion events could not be verified, and thus, no deletions in these genes could be confirmed in the remaining 31 samples.

**Table 2:** Summary of *pfhrp2* and *pfhrp3* genotyping results for the samples selected based on field RDT and microscopy results, and laboratory antigen assay or both

Sample #	Selected on:	Village	Age (yrs)	RDT Results	Microscopy	PET Pf Ct	pAldo^a^ (ng/mL)	pLDH^a^ (ng/mL)	HRP2 (ng/mL)	*pfhrp2* exons 1/2	*pfhrp3* exons 1/2	*pf msp1*^b^	*pf msp2*^b^
					Parasite density (p/µL)								
1	aldo	Chiulu	6	+	256	35.1	43.9	3.7	1.0	+ / +	+ / +		
2	aldo	Chiulu	6	−	0	35.6	40.6	2.7	0.0	+ / +	+ / +		
3	rdt	Chiulu	33	−	80	38.3	0.0	0.0	0.0	+ / +	+ / +		
4	rdt	Herembe	3	−	520	31.8	28.4	2.8	1.0	+ / +	+ / +		
5	ldh	Kasenga	54	+	1717	33.8	26.0	6.0	1.0	+ / +	+ / +		
6	ldh	Kasenga	10	+	1840	33.5	30.6	10.6	1.9	+ / +	+ / +		
7	aldo	Kasenga	6	+	591	35	62.3	6.0	1.7	+ / +	+ / +		
8	aldo	Kasenga	4	+	440	33.9	46.5	4.1	1.4	+ / +	+ / +		
9	rdt	Kasenga	4	−	32	35	0.0	0.0	0.0	+ / +	+ / +		
10	rdt	Kasenga	13	−	96	33.3	0.0	0.0	0.8	+ / +	+ / +		
11	rdt	Kasenga	0	−	221	33.7	0.0	0.0	0.0	+ / +	+ / +		
12	ldh aldo	Katale	2	+	480	31.8	360.9	114.6	21.2	+ / +	+ / +		
13	ldh aldo	Katale	4	+	2628	28.7	797.1	214.5	42.4	+ / +	+ / +		
14	ldh aldo	Katale	10	−	0	35.3	76.4	8.9	0.2	+ / +	+ / +		
15	ldh aldo rdt	Katale	9	−	2000	28.2	210.8	62.4	5.0	+ / +	+ / +		
16	aldo rdt	Katale	4	−	280	31	44.1	0.0	0.0	+ / +	−/−	−/−	+ / −
17	ldh aldo	Kigege	2	+	48	32.4	190.5	80.3	12.4	+ / +	+ / +		
18	rdt	Kigege	38	−	16	38.9	0.0	0.0	0.0	+ / +	−/ +	−/ +	−/−
19	rdt	Kigege	8	−	520	34.8	0.0	0.0	0.0	+ / +	+ / +		
20	ldh	Kitunguli	6	+	0	31.5	42.9	39.2	1.5	+ / +	+ / +		
21	rdt	Mkowela	43	-	440	37.3	0.0	0.0	0.0	+ / +	±	+ / −	−/ +
22	aldo	Mkowela	2	+	0	35.3	51.0	5.4	1.3	+ / +	+ / +		
23	ldh aldo	Mkowela	11	−	0	35.8	46.3	5.6	0.0	+ / +	+ / +		
24	aldo	Mkunwa	3	+	48	36	33.0	5.9	0.1	+ / +	+ / +		
25	rdt	Mtawarawa	14	−	176	33.2	0.0	0.0	0.0	+ / +	+ / +		
26	rdt	Mtawarawa	22	−	96	34.9	0.0	0.0	0.1	+ / +	+ / +		
27	ldh	Mwamila	2	+	11,480	27.6	338.3	159.4	35.5	+ / +	+ / +		
28	ldh aldo rdt	Nyangalamila	8	−	288	34	54.7	11.0	0.0	+ / +	+ / +		
29	ldh aldo	Nyankoronko	7	+	104,000	25.7	760.1	242.9	37.5	+ / +	+ / +		
30	ldh aldo	Nyankoronko	2	+	160	33.8	98.8	18.3	2.1	+ / +	+ / +		
31	ldh aldo	Nyankoronko	4	+	28,800	29.8	76.2	19.1	0.9	+ / +	+ / +		
32	ldh	Nyankoronko	6	+	440	36.5	24.2	7.9	1.4	+ / −	+ / +	+ / −	+ / −
33	aldo rdt	Nyankoronko	43	−	48	37	31.3	0.0	0.0	+ / +	+ / +		
34	rdt	Nyankoronko	10	−	760	31.3	0.0	0.0	0.8	+ / +	+ / +		
35	ldh rdt	Nyankoronko	9	−	88,600	25.5	187.5	85.1	16.6	+ / +	+ / +		

^a^Antigens listed indicate aberrant relationship between HRP2 and that antigen’s assay signal; RDT indicates microscopy/RDT discordance

^b^Genotyping for *pfmsp1* and *pfmsp2* not performed if the sample showed amplification of all *pfhrp2* and *pfhrp3* targets

## Discussion

The samples used in this study were collected during a community-based survey which was conducted in four regions with persistently high malaria burden over the past 10yrs and used to assess the presence and prevalence of *pfhrp2* and *pfhrp*3 gene deletions in Tanzania. The findings from field RDT and microscopy tests as well as the laboratory multiplex antigen test indicate that the vast majority of *P. falciparum* infections in Tanzania produced high levels of HRP2 (and HRP3) antigens, which would be recognized by HRP2-based RDTs. Although RDTs did not detect some infections from persons confirmed to be *P. falciparum* positive by PCR, the evidence presented here suggests that these false negative RDT results were not due to *pfhrp2* and *pfhrp3* gene deletion.

During the survey, extensive efforts were taken to protect the quality of the RDTs. The experienced study team ensured that RDTs were stored in appropriate conditions as per the manufacturers’ instructions; transported in good conditions (ambient temperature with minimal humidity) and the tests were also performed by experienced technicians. To minimize operator errors, the testing process and reading of RDT results were done in the presence of other members of the team who ensured that any doubtful RDTs results were correctly read and interpreted. Studies conducted elsewhere reported that the performance of HRP2-based RDTs depends on the level of parasitaemia [1, 8, 26], with the lower limit of detection generally around 200 parasites/µL [1]. This community survey included mainly asymptomatic persons, and some had low-level parasitaemia by microscopy (bellow the detection limits of RDTs); this could possibly explain some of the discordances between the field RDT results and the laboratory antigen test. In total, 1998 (27.3% of all) participants were concordant between those two tests, whereas 372 (6.0%) individuals were positive for RDTs alone and 325 (4.4%) were positive only by the bead-based multiplex assay. As RDTs are designed for reliable detection of parasite densities more typical of clinical relevance (200p/µL or greater), their use in asymptomatic population may miss low-density infections with low levels of HRP2 antigens [48]. Even in this community setting, it was encouraging to see that RDTs were able to detect the majority of persons with malaria antigenemia as determined by the bead-based multiplex assay. Of the 2323 total samples found to be antigen positive, 1998 (86.0%) came from persons who were RDT positive. Considering the generally low parasite densities for any *P. falciparum-*infected persons in this survey, concordance was also good for all three tests; microscopy, RDTs, and the laboratory antigen test. Of the 1158 total microscopy positives, 1063 (91.8%) were also RDT positive, and 1050 (90.7%) were also positive by the bead-based multiplex assay. Concordance among multiple malaria indicators provides greater confidence for the true levels of malaria in a populace.

Of the 35 samples selected for further molecular investigation (from asymptomatic individuals), which were positive for *P. falciparum* by PCR, the majority (27/35) had relatively low parasite densities ranging from 0 to 1000 parasite/µL. However, eight samples had higher parasite densities (> 1000 asexual parasites/µl), and two of these had very high parasite density (88,600 and 104,000 parasite/µL, respectively). Two of these eight persons gave negative RDT results which could potentially be explained by prozone effect where an excess of antigen leads to false-negative results [49]. All eight of these higher density infections were found to have detectable HRP2 antigen, though at much lower blood concentrations than typical given those levels of *P. falciparum* parasite densities.

It was surprising to find that 16 out of the 35 samples which were both microscopy and PCR positive with intact *pfhrp2* and *pfhrp3* gene, had no or atypical antigen levels. Of these, 7 did not have antigen completely and 9 had atypical relationship between the pan*-Plasmodium* markers and HRP2. The absence of detectable HRP2 antigens in these samples can possibly be due to deterioration/degradation of the antigens, which were analysed after DBS storage for more than 7 months. However, more detailed assessments of such samples might be needed to determine the reasons for such discordant results.

The multiplex antigen screening allowed for reconfirmation of HRP2 (and possibly HRP3) antigen profiles for DBS samples. Of all 7543 samples screened by the multiplex antigen test, few (28, 0.4%) samples had a complete absence of HRP2 or an aberrant relationship between the assay signal for the pan markers and HRP2. This could be due to very low parasite densities or infections with non-*P. falciparum* species, but this observation could not be explained by deletions of the *pfhrp2* and *pfhrp3* genes.

This report adds to the literature in the same way as studies in Honduras [47] and French Guiana [50], where no deletion of *pfhrp2* gene were reported. However, these findings are in contrast with the results from two recent studies which were conducted in Mbeya, Mtwara, Mwanza and Bagamoyo in Tanzania using samples collected in 2010 and between 2016 and 2018, respectively. The studies found evidence of sporadic occurrence of *pfhrp2 and pfhrp3* gene deletion in some areas, with 1.7% of isolates tested reported to have a deletion of either of the genes [30], and 0.7% carried *pfhrp2* deletions while another 0.7% carried a deletion of *pfhrp3* [31]. Though recommended molecular tests [32] were used in the previous studies, the overall sample sizes were very small with only 176 and 149 Tanzanian samples tested in the two studies, respectively [30, 31]. Malaria endemicity and sampling method could potentially explain the differences between the present study which sampled mainly asymptomatic persons at the community) [33] and the previous studies which sampled symptomatic patients at health facilities [30, 31]. Despite very low estimates of the prevalence of single or double deletions of the genes in the previous studies, there is a potential that those parasite strains are still present at very low-levels in these regions. Thus, future studies will be required to cover these and other areas with varying epidemiological profile and using the World Health Organization (WHO) recommended protocol to confirm the absence or presence of the deletions.

A limitation of the current study is that, the samples used were from a community survey which enrolled mainly asymptomatic individuals and sampling was not done using the protocol recommended by the WHO for *pfhrp2/3* gene deletion investigation. The WHO recommends that individuals seeking care for febrile illness at health facilities should be selected as a target population for the investigation of *pfhrp2* and/or *phrp3* gene deletions. This is because of the increased chances of detecting parasitaemia in symptomatic patients rather than asymptomatic persons. Due to this limitation, these findings are not conclusive rather they provide baseline information of *pfhrp2/pfhrp3 gene* deletion, and thus needs further studies. Also with the use of multiplex antigen assay, a single gene deletion of *pfhrp2* or *pfhrp3* alone could be masked by a parasite producing at least one of these antigens. Deletions (or loss-of-function mutations) in both genes would lead to a complete absence of HRP2/HRP3 antigens in a *P. falciparum* infection. However, if one of these antigens was being produced by the parasites, the algorithm defined here may have not been able to identify such blood samples as suspicious if the assay signal for HRP2 remained high. Additionally, the molecular tests performed here were only for complete gene deletions, and any loss-of-function by point mutations leading to antigen non-expression would not be captured since the *pfhrp2* or *pfhrp3* specific DNA amplification would still occur using the current protocol. Another limitation from the study is that it does not provide estimates for the entire country, since only 4 out of the 26 regions of mainland Tanzania were included in this study. In addition, these regions have high malaria transmission where parasite mutations including *pfhrp2*/3 gene deletion could be attenuated by high recombination rates involving different strains, which occur in such areas. Further studies will need to be conducted on *P. falciparum* isolates collected from symptomatic patients and other geographical regions of Tanzania (especially in low transmission areas) in order to increase the chances of detecting *pfhrp2* and *pfhrp3* gene deletion in different parts of the country.

## Conclusions

Though a low number of false negative RDT results were found in Tanzania, these could not be explained by *pfhrp2* or *pfhrp3* gene deletions. However, single gene deletions could have been missed by the multiplex antigen assay suggesting parasites with deletion of one of the genes might be circulating in the population. Overall, the study results suggest that HRP2-based RDTs for detection of *P. falciparum* infection and confirmatory diagnosis of malaria in the surveyed area in Tanzania can be used as a reliable tool for malaria case management. There is a need for continued surveillance to monitor the status of *pfhrp*2 and/or *pfhrp3* deletions in the future.

## Supplementary information

**Additional file 1: Table S1.** Primers and PCR reaction conditions to amplify *pfhrp2* and *pfhrp3* genes. **Table S2.** Primers and PCR reaction conditions to amplify *msp-1* and *msp-2* genes

## Data Availability

The datasets generated and/or analysed during the current study are available from the corresponding author on reasonable request.
