# Supplementary material for: Community-based surveys for Plasmodium falciparum pfhrp2 and pfhrp3 gene deletions in selected regions of mainland Tanzania
Source: Malar J. 2020 Nov 4;19:391. doi: 10.1186/s12936-020-03459-3 (PMC7640459; doi:10.1186/s12936-020-03459-3)
Supplement: Supplementary file 1 — Additional file 1: Table S1. Primers and PCR reaction conditions to amplify pfhrp2 and pfhrp3 genes. Table S2. Primers and PCR reaction conditions to amplify msp-1 and msp-2 genes [file 12936_2020_3459_MOESM1_ESM.docx]

**Additional file 1: Table S1. Details of Primers and PCR reaction conditions to amplify *pfhrp2* and *pfhrp3* genes**


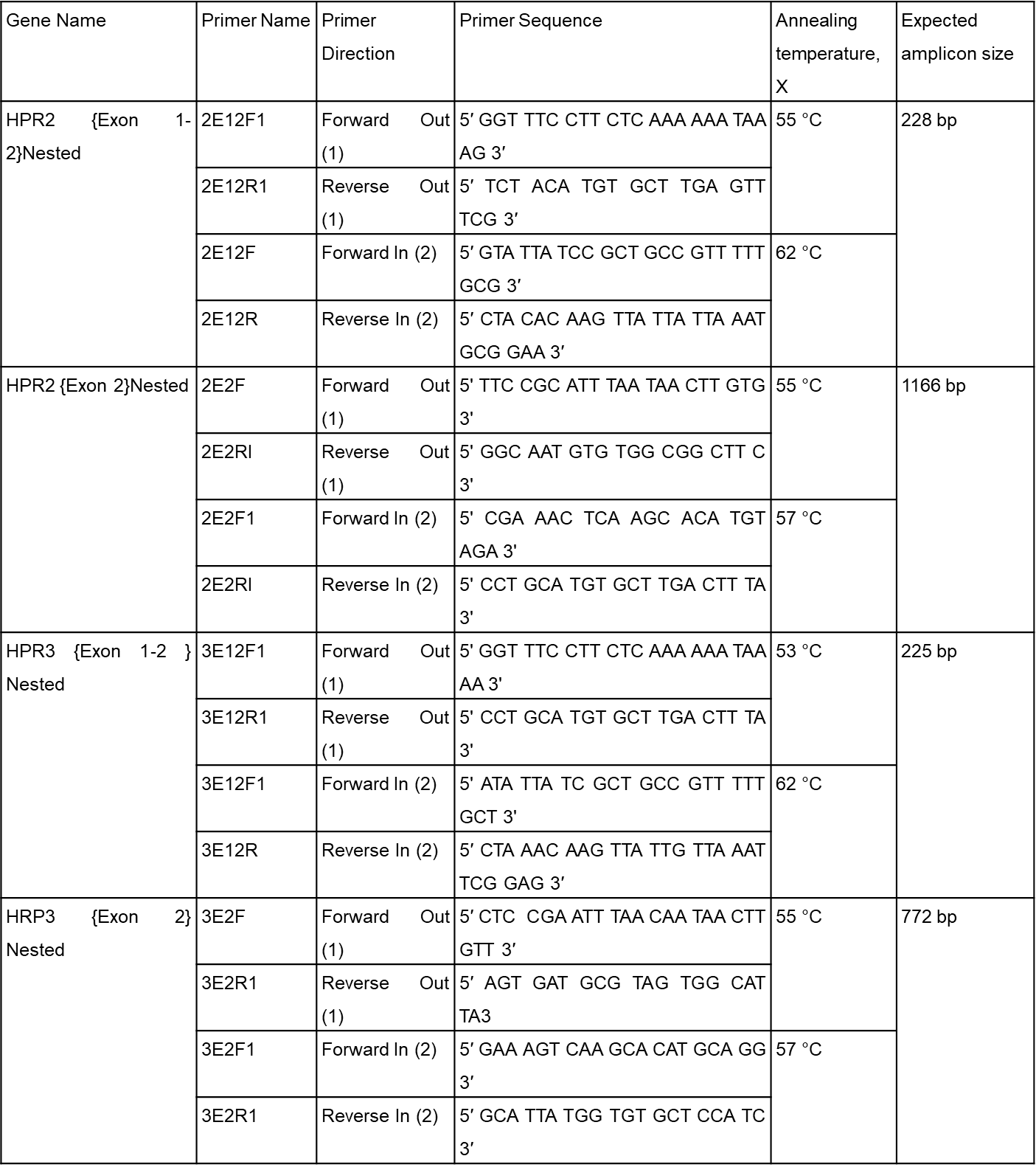


All reactions were conducted using the following reaction condition: 95°C/5min – [95°C /30sec/X /30sec/68°C /30 sec] x30cycles – 68°C /5min – 4°C /infinity

**Additional file 1: Table S2. Details of Primers and PCR reaction conditions used to amplify *msp-1* and *msp-2* genes**

**
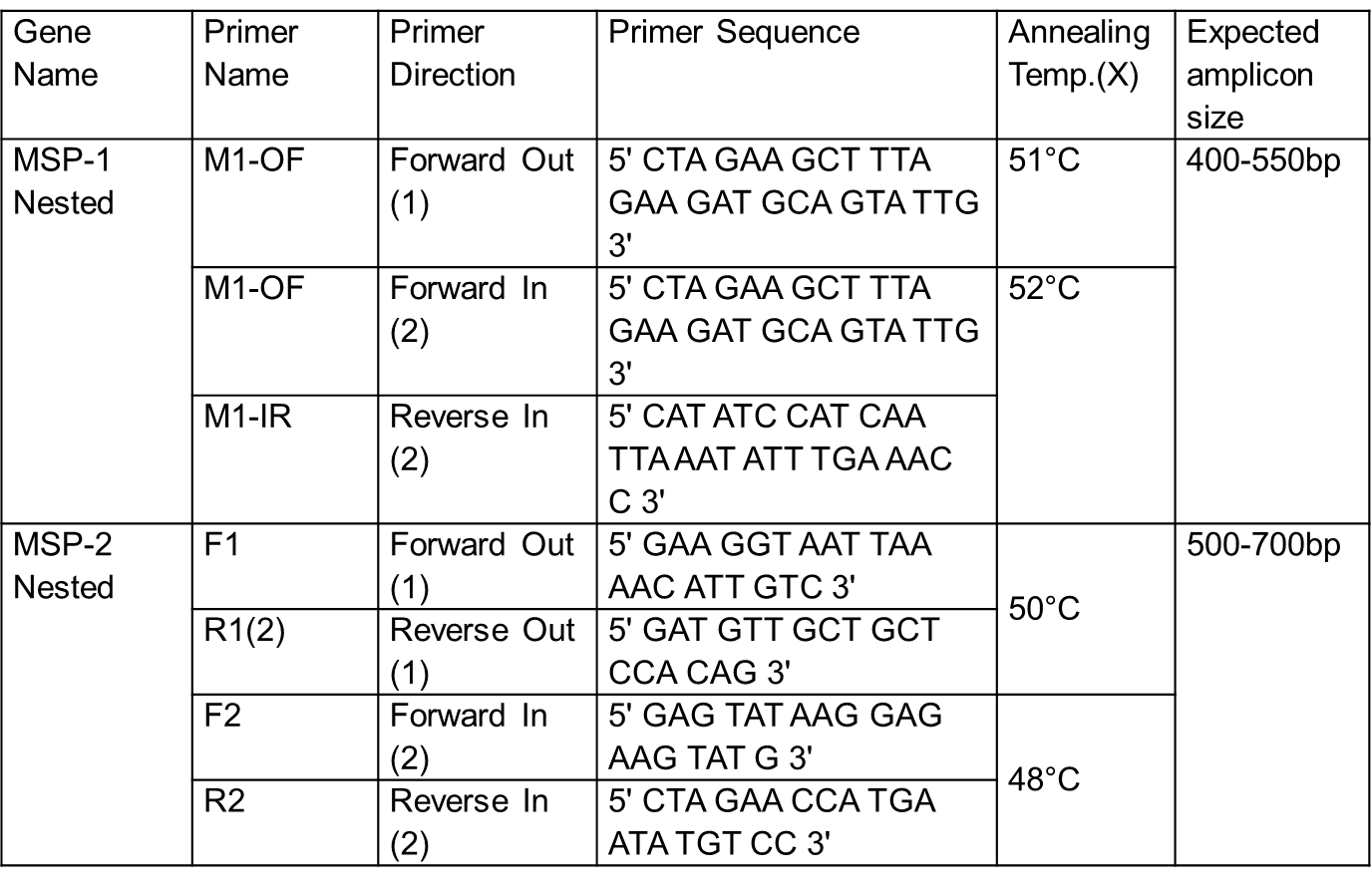
**

All reactions were conducted using the following reaction condition: 95°C/5min – [95°C /30sec/X /30sec/68°C /1 min] x30cycles – 68°C /5min – 4°C /infinity
